# Comparative physiological and transcriptomic analysis of two salt-tolerant soybean germplasms response to low phosphorus stress: role of phosphorus uptake and antioxidant capacity

**DOI:** 10.1186/s12870-023-04677-y

**Published:** 2023-12-20

**Authors:** Xiu-Wen Zhou, Xing-Dong Yao, De-Xin He, He-Xiang Sun, Fu-Ti Xie

**Affiliations:** Soybean Research Institute, Shenyang Agricultural University, Shenyang, China

**Keywords:** Soybean (*Glycine max*), Root, Transcriptome analysis, Phosphorus, Salt

## Abstract

**Background:**

Phosphorus (P) and salt stress are common abiotic stressors that limit crop growth and development, but the response mechanism of soybean to low phosphorus (LP) and salt (S) combined stress remains unclear.

**Results:**

In this study, two soybean germplasms with similar salt tolerance but contrasting P-efficiency, A74 (salt-tolerant and P-efficient) and A6 (salt-tolerant and P-inefficient), were selected as materials. By combining physiochemical and transcriptional analysis, we aimed to elucidate the mechanism by which soybean maintains high P-efficiency under salt stress. In total, 14,075 differentially expressed genes were identified through pairwise comparison. PageMan analysis subsequently revealed several significantly enriched categories in the LP vs. control (CK) or low phosphorus + salt (LPS) vs. S comparative combination when compared to A6, in the case of A74. These categories included genes involved in mitochondrial electron transport, secondary metabolism, stress, misc, transcription factors and transport. Additionally, weighted correlation network analysis identified two modules that were highly correlated with acid phosphatase and antioxidant enzyme activity. Citrate synthase gene (*CS*), acyl-coenzyme A oxidase4 gene (*ACX)*, cytokinin dehydrogenase 7 gene (*CKXs*), and two-component response regulator ARR2 gene (*ARR2*) were identified as the most central hub genes in these two modules.

**Conclusion:**

In summary, we have pinpointed the gene categories responsible for the LP response variations between the two salt-tolerant germplasms, which are mainly related to antioxidant, and P uptake process. Further, the discovery of the hub genes layed the foundation for further exploration of the molecular mechanism of salt-tolerant and P-efficient in soybean.

**Supplementary Information:**

The online version contains supplementary material available at 10.1186/s12870-023-04677-y.

## Background

Salinity stress and phosphorus (P) deficiency are two of the most significant soil limitations to crop production. Furthermore, salinity hinders the absorption and transportation of mineral nutrients, thereby exacerbating the restriction of nutrient availability for plants [1]. Consequently, crops cultivated in saline soil experience several abiotic stress factors concurrently. Plants that survive in such challenging environments are compelled to develop adaptive mechanisms.

P is an irreplaceable macronutrient and an essential element for the growth and development of plants. It is a crucial component of basic biomolecules and plays a vital role in various cellular activities [2–4]. Approximately 40% of the world’s arable land is deficient in effective P [5], and low P stress is one of the most common biological stresses in agricultural ecosystems [6]. The situation is even worse in China, where 74% of arable land is effective P deficient and more than 30% of arable land requires P fertilizer to meet crop nutrient needs [7]. P is a non-renewable resource and consumed quickly [8], P deficiency resulted in crop reduction in yield to 30–40% [9]. Therefore, it is crucial to enhance the absorption and utilization efficiency of P in crops to reduce dependence on natural resources and minimize the adverse environmental impacts of excessive use of P fertilizers [10]. Plants have evolved various mechanisms to cope with persistent P deficiency, such as altering their root system architecture, boosting acid phosphatase (ACP) activity, secreting small molecular organic acids, activating P-responsive genes, and establishing symbiotic relationships with mycorrhizal fungi [11, 12]. Plants respond to P deficiency by forming long, slender roots, increasing lateral root production and growth, developing cluster roots, and increasing root hair length and density [11, 13–15]. Plants secrete or release organic acids, such as citric acid, malic acid, and oxalic acid to response to P-deficiency and these organic acids dissolved phosphate in the soil and converted it into soluble P that is available for plant uptake [16]. In addition, plants also produce acid phosphatases, which promoted the release and conversion of phosphate, thereby improving the efficiency of P uptake by plants [17].

Globally, the area of saline soils is increasing at a rate of 1.0 to 1.5 million hectares per year, and currently, salinity affects 10% of the world’s arable land [18]. In China, the widespread distribution of saline soils, which covers about 99.13 million hectares, is a significant factor limiting crop yields [19]. The adverse effects of salt stress on plants include osmotic stress, ion toxicity, and nutrient imbalance caused by interference with nutrient uptake and transport [20]. Salt stress affects the uptake of P by plants. Studies demonstrated that plants experience a decrease in P concentration under salt stress, particularly in high-salt environments. This reduction in P levels was observed in both plant leaves and roots. This was because salt stress causes a decrease in the growth and uptake capacity of plant roots, thus reducing the uptake of P by plants [21, 22]. In addition, salt stress affects the effectiveness of P in the soil, making it more difficult for plants to absorb P from the soil [23]. For instance, salinity stress decreased the efficiency of P uptake in crops such as chickpea (*Cicer arietinum*) [24]. Previous research indicated that the impact of simultaneous P deficiency and salt stress on physiological markers closely resembled that of plants exposed to salt stress alone, and with a more pronounced effect on the root system [25]. The inhibition of resource allocation in plants was even more pronounced and detrimental to maize under the combined stress of salt and low P, as compared to low P stress alone [26]. Therefore, how to improve the P uptake efficiency of crops in high salt soil is a problem worthy of our attention.

Soybean holds a significant economic value and demonstrates a moderate tolerance to low P and salt [27]. The plant obtains P through its root system and transports it to the shoots which is essential for metabolic processes such as cell synthesis, photosynthesis, and respiration [28]. Previous studies focused on the adaptation mechanism of soybean under low P or salt stress without considering their combined effects [1]. The aim of this study was to investigate the molecular mechanism of soybean that maintain P-efficiency under salt stress. Two soybean germplasms, A6 and A74, were selected on the basis of their similar salt-tolerance and contrasting P efficiencies. The growth and physiological parameters of these two soybean germplasms were assessed under conditions of low P and salt stress. Additionally, genome-wide transcriptome analyses were conducted to identify differentially expressed genes. Furthermore, PageMan and WGCNA analyses were conducted to identify pathways and hub genes related to the high P-efficiency under salt stress. The findings of this study enhance our comprehension of the molecular mechanism underlying P-efficiency under salt stress in soybean and facilitate the development of crops with improved growth and development under these conditions.

## Results

### Effects of LP and S alone or combination on the growth and physiological characteristics of two soybean germplasms

**Fig. 1:**
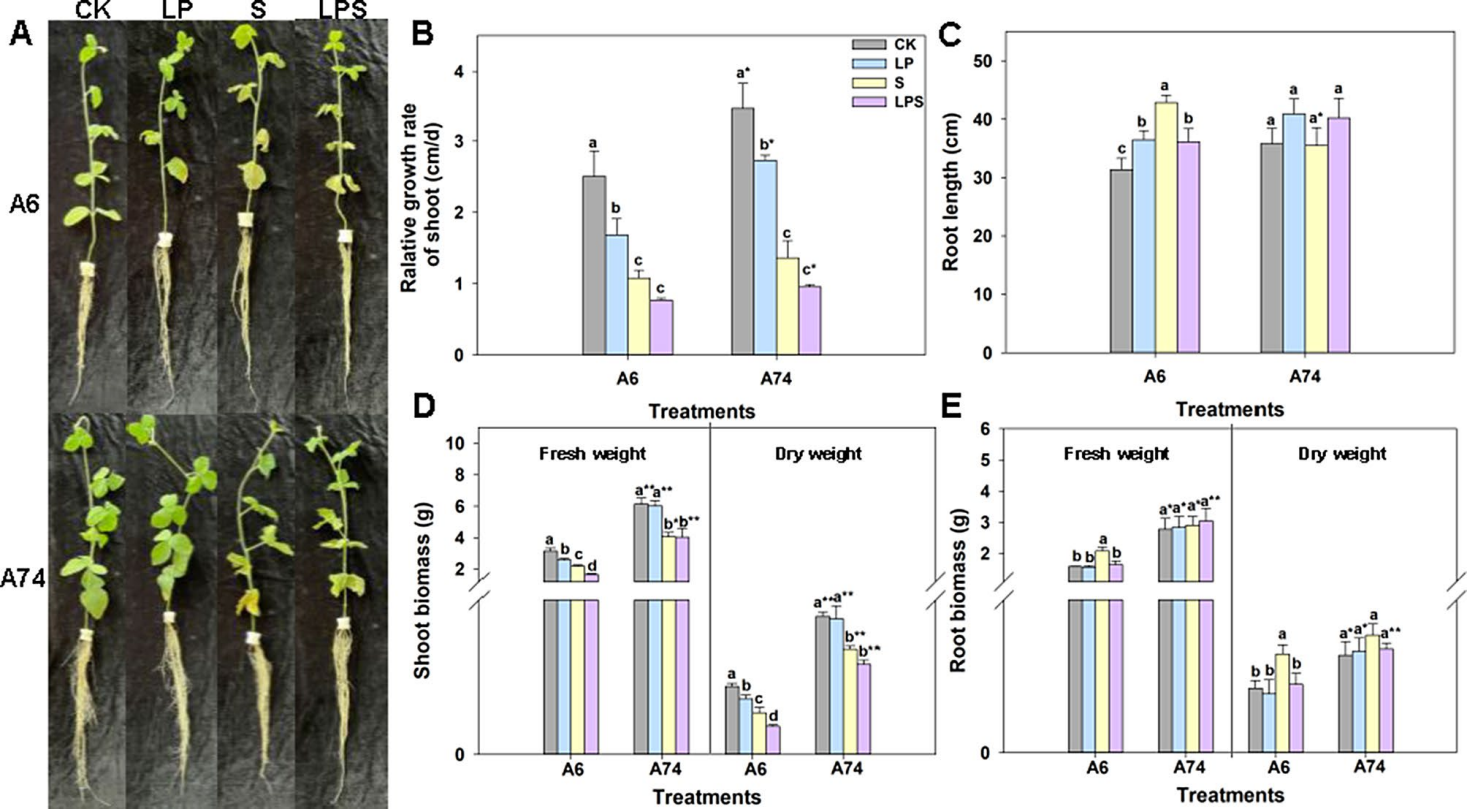
Soybean phenotypic features under each treatment. Soybean seedlings were treated at V2 growth stage, which represented second vegetative stage of the soybean’s development and typically occurred when the soybean plant has developed two fully expanded trifoliate leaves. Growth status of two soybean germplasms (**A**) was photographed after fourteen days, relative growth rate of shoot (**B**), root length (**C**), shoot biomass (**D**) and root biomass (**E**) were measured after the same period. Different letters above columns of the different color indicate statistic significant difference at *P* < 0.05 (Duncan’ s multiple range test). The * (*P* < 0.05) and ** (*P* < 0.01) indicate significant differences between bars of the same color (Student’s t-test). CK represents control, LP represents low phosphorus, S represents salt and LPS represents low phosphorus + salt

The impact of low phosphorus (LP) and salt (S) alone or combination stress on the growth and physiological characteristics of two soybean germplasms were investigated. The results showed that A74 exhibited superior growth and development under LP and low phosphorus + salt (LPS) treatment compared to A6 (Fig. 1A). Besides, in LP environment, the growth of A74 was not affected, and A6 significantly deteriorated. The relative growth rate of shoot of both germplasms was significantly decreased under all three stress conditions compared to the control, but A74 increased significantly under LP and LPS treatments compared to A6 (Fig. 1B). Under stress, the root length of A6 significantly extended compared to the control, while there was no significant difference for A74 (Fig. 1C). The shoot biomass of A6 was significantly reduced under LP treatment compared to the control, while A74 was unaffected. Similarly, the shoot biomass of A6 was significantly reduced under LPS treatment compared to S treatment alone, but no significant difference was observed for A74 (Fig. 1D). The root biomass of A6 increased significantly under S treatment compared to the control. For all stress treatments, no difference was observed in the root biomass of A74, however, its root dry weight was significantly higher than that of A6 under LP and LPS treatments (Fig. 1E). In summary, A74 outperformed A6 significantly when exposed to LP or LPS stress.

### Effects of LP and S alone or combination on roots of soybean seedling

**Fig. 2:**
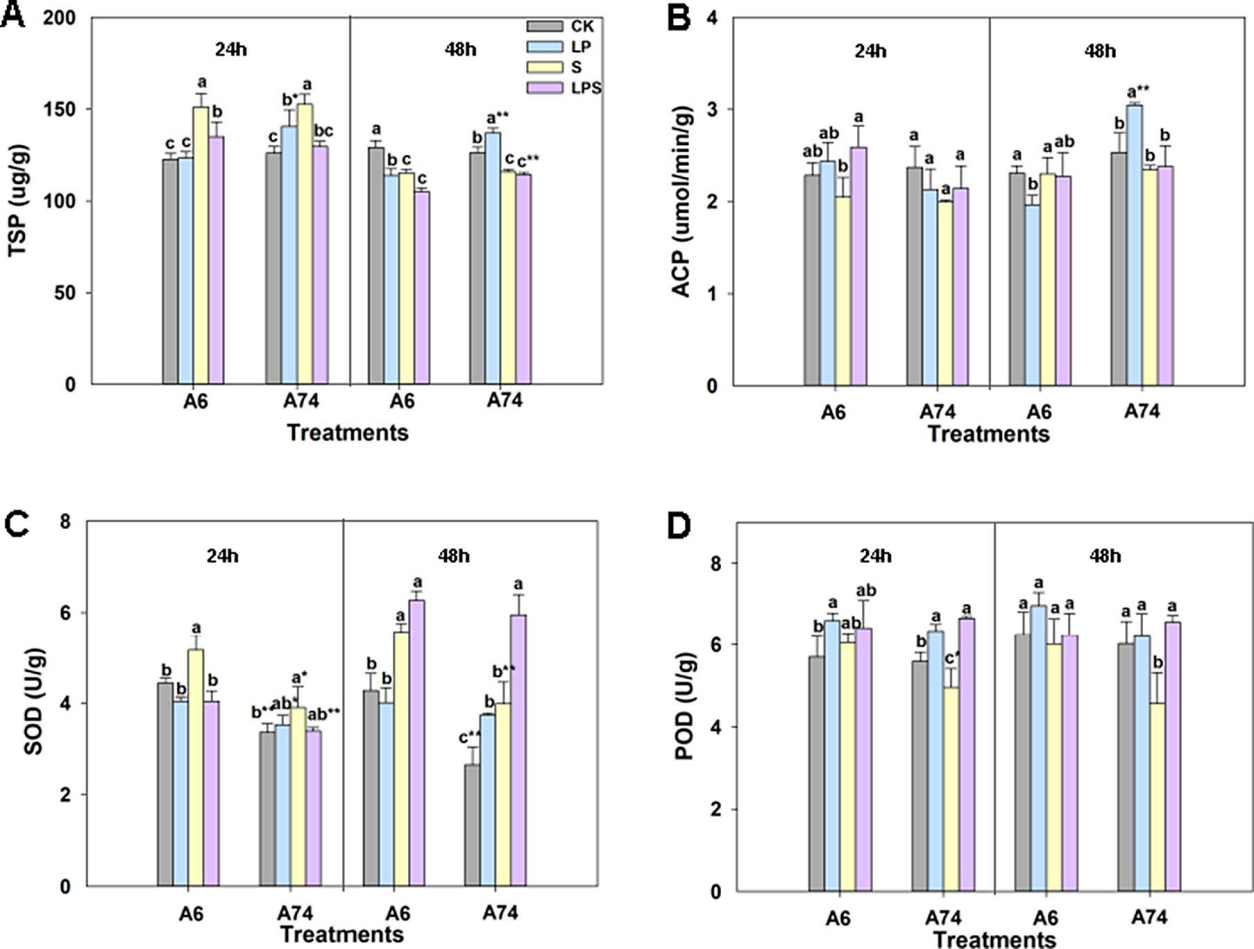
The root resistance indexes of two soybean germplasms were measured under four different treatments. The soybeans were cultured to the V2 growth stage before being transferred to hydroponic cultures that mimicked CK (control), LP (50 µM H_3_PO_4_), S (200 mM NaCl), and LPS (50 µM H_3_PO_4_ and 200 mM NaCl) conditions for 24 and 48 h. The levels of TSP content (**A**), ACP activity (**B**), SOD activity (**C**), and POD activity (**D**) in roots were measured. Different letters above columns of different colors indicate statistically significant differences at *P* < 0.05 (Duncan’s multiple range test). The * (*P* < 0.05) and ** (*P* < 0.01) indicate significant differences between bars of the same color (Student’s t-test). CK represents control, LP represents low phosphorus, S represents salt and LPS represents low phosphorus + salt

**Fig. 3:**
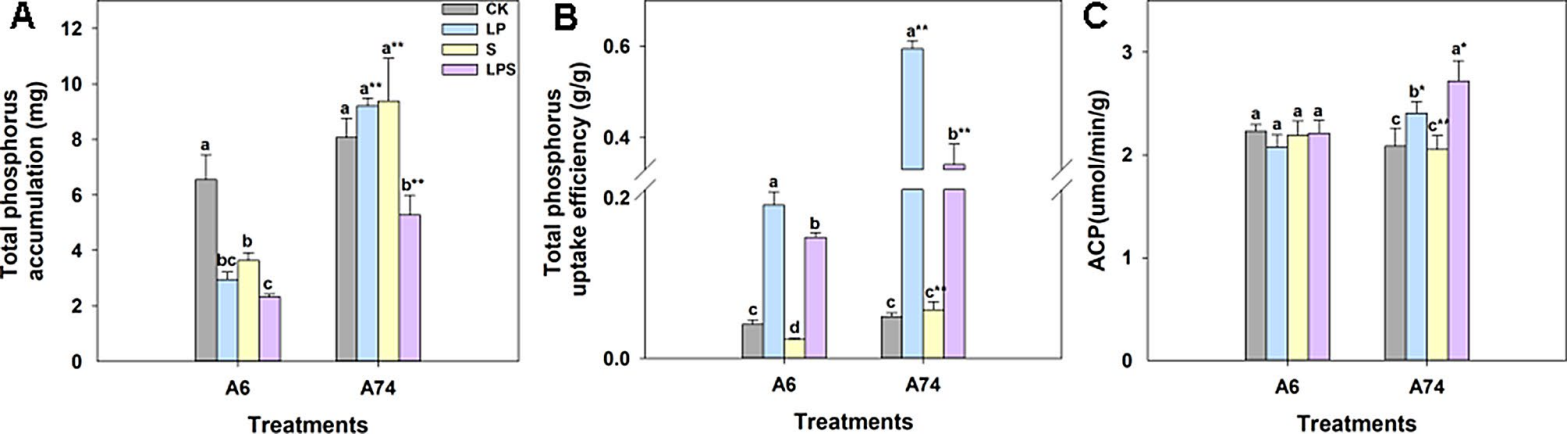
The P concentration of each plant was determined after 14 days of treatment during the V2 stage. The bar chart shows the average total P accumulation (**A**), total P uptake efficiency (**B**) and ACP of roots (**C**) of two soybean germplasms under each treatment, with three replicates per treatment. Different letters above columns of the different color indicate statistic significant difference at *P* < 0.05 (Duncan’ s multiple range test). The * (*P* < 0.05) and ** (*P* < 0.01) indicate significant differences between bars of the same color (Student’s t-test). CK represents control, LP represents low phosphorus, S represents salt and LPS represents low phosphorus + salt

To study the response in the roots of A6 and A74 under LP and S alone or combination stress, the soybean seedlings were treated for 24 and 48 h respectively. The roots samples were collected for physiological analysis. After 24 h of treatment, A6 did not show a significant difference in total soluble protein (TSP) content under LP stress compared to the control. However, A74 exhibited a significant increase in TSP content, with TSP levels being significantly higher in A74 than in A6 (Fig. 2A). Following 48 h of treatment, all stress conditions significantly reduced TSP content of A6 when compared to the control. Meanwhile, TSP content of A74 significantly increased under LP treatment, and was significantly higher than A6’s under both LP and LPS treatment as shown in Fig. 3A. At 48 h, acid phosphatase (ACP) activity of A6 significantly decreased under LP treatment compared to the control, whereas A74’s significantly increased. Moreover, A74 alone exhibited a significant increase in ACP activity under LP conditions compared to A6 (Fig. 2B). After 48 h of treatment, superoxide dismutase (SOD) activity in A74 under LP treatment was significantly higher than that in the control group. Moreover, SOD activity in A74 under LPS treatment was significantly higher than that under S treatment. However, no such phenomenon was observed in A6 (Fig. 2C). Compared to S treatment, peroxidase (POD) activity of A74 was significantly increased at 24 and 48 h under LPS treatment, while no significant changes were observed in A6 (Fig. 2D). These results demonstrated that A74 exhibits a stronger resistance to LP and LPS stresses than A6.

### Differential P response in two soybean germplasms under different stress conditions

Under all stress conditions, the total P accumulation of A6 was significantly decreased compared to the control. The greatest decrease was observed under LPS treatment. On the other hand, A74 showed a significant decrease in total P accumulation only under LPS treatment, while LP and S treatment alone had no effect on it (Fig. 3A). The trend of P accumulation of shoots was consistent with the results mentioned above (Fig. S1A). Under stress treatments, the root P accumulation of A74 was significantly higher than that of A6 (Fig. S1B). Total P uptake efficiency of A74 was significantly increased under all stresses, compared to A6 (Fig. 3B). The trend of P absorption efficiency in both aboveground and root parts was similar (Fig. S1C-D). ACP activity of A6 did not change significantly under stress conditions, whereas A74 showed a significant increase in ACP content under LP and LPS treatments. A74 also had significantly higher ACP activity than A6 under three stress conditions (Fig. 3C). These results suggest that A74 was a P-efficient germplasm compared to A6, with ACP playing a crucial role in this process.

### Identification of differentially expressed genes

**Fig. 4:**
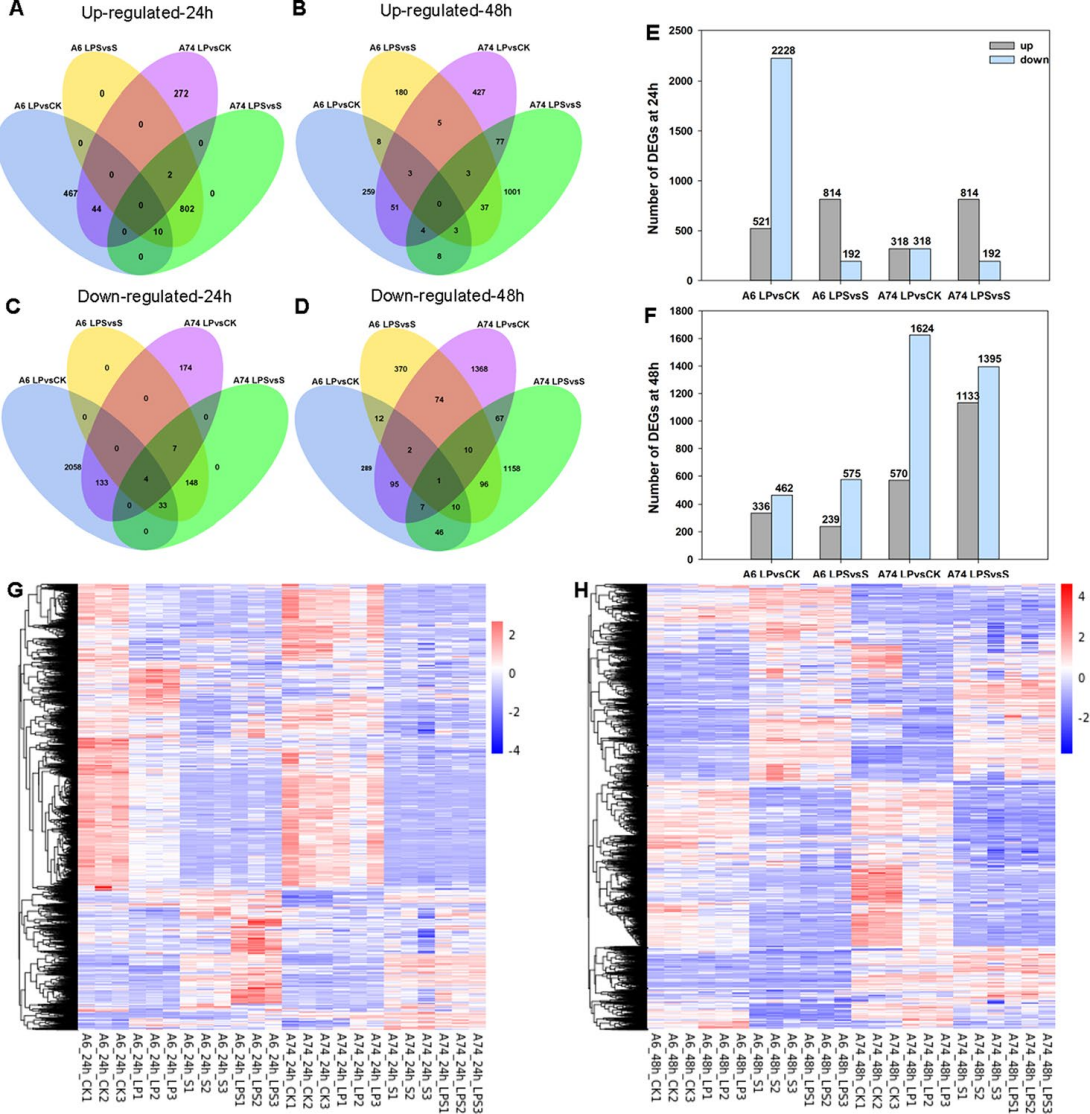
Summary of different expression genes after exposed to different treatment condition. Venn diagram showing the overlap of up-regulated genes at 24 h (**A**), 48 h (**B**) and down-regulated genes at 24 h (**C**), 48 h (**D**). The number DEGs under different treatment at 24 h (**E**), 48 h (**F**). Heatmap of the relative expression levels of DEGs under different treatment at 24 h (**G**), 48 h (**H**). CK represents control, LP represents low phosphorus, S represents salt and LPS represents low phosphorus + salt

To further explore the molecular mechanism of A6 and A74 under LP and S alone or combination stress, we conducted whole-genome transcriptome analysis on the roots of A6 and A74 under CK, LP, S, and LPS conditions. Comparisons of A6 LP vs. CK, A6 LPS vs. S, A74 LP vs. CK and A74 LPS vs. S consisted of 521, 814, 318, 814 up-regulated genes and 2,228, 192, 318, 192 down-regulated genes at 24 h, respectively. At 48 h, there were 336, 239, 570, 1,133 up-regulated genes and 462, 575, 1,624, 1,395 down-regulated genes, respectively (Fig. 4A-F). Notably, comparisons of A74 LP vs. CK showed 318 up-regulated genes at 24 h, of which 2 genes were also up-regulated in the A74 LPS vs. S (Fig. 4A). At 48 h, there were 84 co-upregulated genes in the two comparative combinations (Fig. 4B). 2,228 down-regulated genes in the A6 LP vs. CK comparative combination at 24 h, of which 37 genes were also down-regulated in A6 LPS vs. S (Fig. 4C), and 25 genes were co-downregulated in both comparative combinations at 48 h (Fig. 4D). At 24 h, A6 exhibited a greater number of DEGs compared to A74 in the LP vs. CK comparison, indicating A6 was sensitive to LP conditions and responded early to stress. Conversely, at 48 h, A74 displayed a higher DEGs count than A6 in both the LP vs. CK and LPS vs. S comparison, implying that A74 exhibited a delayed yet more enduring response to LP-related stress (Fig. 4A-F). Moreover, the overall expression pattern was visualized in a heat map (Fig. 4G,H), which provided an overview of the changes in gene expression.

### Functional categorization of the deferentially expressed genes

**Fig. 5:**
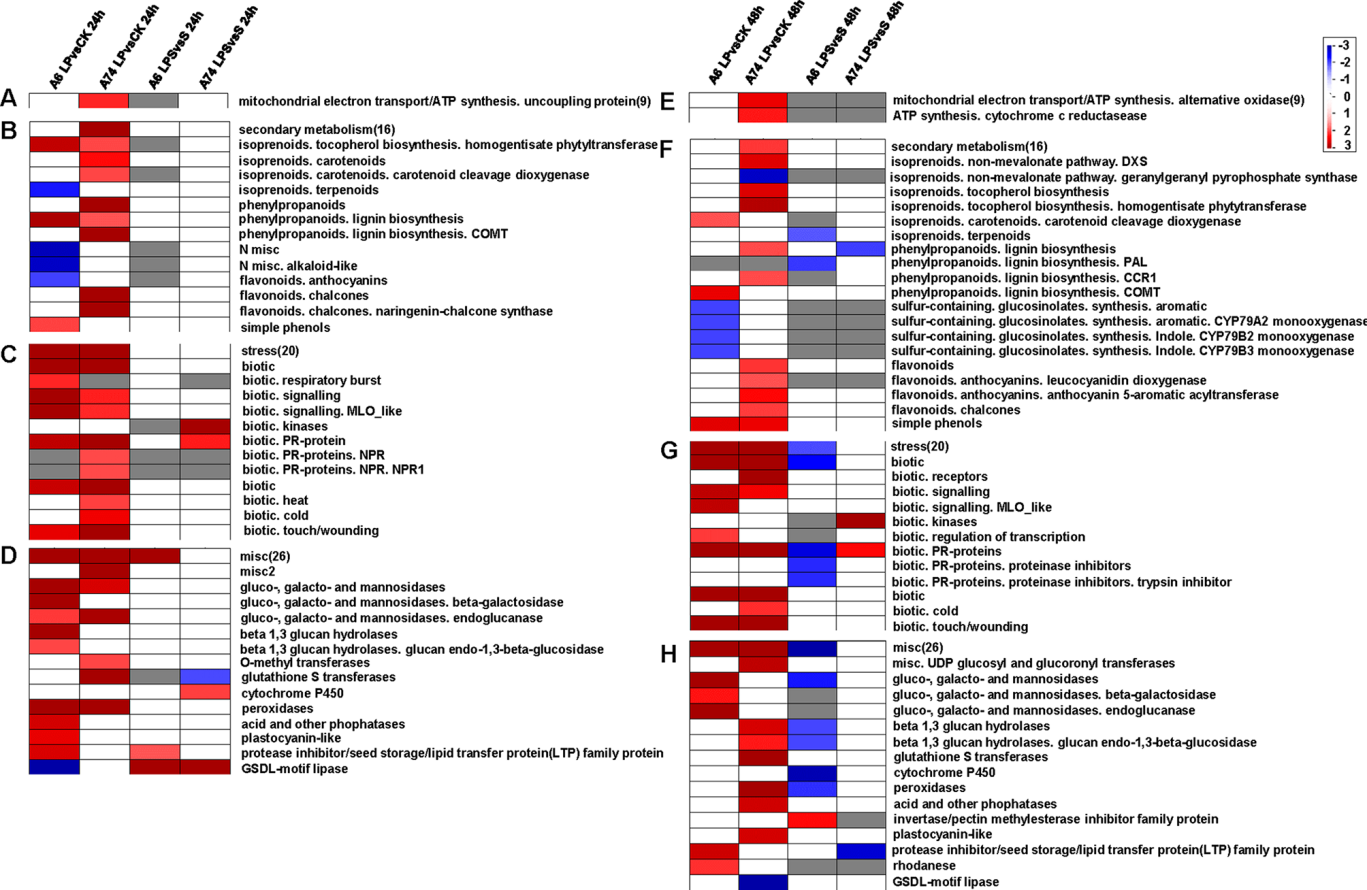
PageMan display of coordinated changes of selected gene categories activated by low phosphorus and salt. Mitochondrial electron transport (**A**, **E**), secondary metabolism (**B**, **F**), stress (**C**, **G**), misc (**D**, **H**). The log2foldchange of A6 LP vs. CK, A74 LP vs. CK, A6 LPS vs. S and A74 LPS vs. S were subjected to over-representation analysis. Red color is significant enrichment of up-regulated genes; blue color is significant depletion of up-regulated genes. The complete analysis and its display are provided in Supplemental Figure S3 and Table S1

To analyze the relationship between the enriched transcripts of different treatments, the PageMan analysis generated clusters for all annotated DEGs. We focused on bins involved in mitochondrial electron transport (9), secondary metabolism (16), stress (20), and misc (26) for this analysis (Fig. 5, Fig S3 and Table S1). In addition, we focused on the DEGs situation of transcription factors and transporters (Fig. S2). ATP synthesis-related sub-bins were only up-enriched in the A74 LP vs. CK comparison (Fig. 5A and E). Additionally, flavonoid-related genes were significantly up-enriched in the same comparison combination at both 24 and 48 h (Fig. 5B and F, Table S1). Kinase-related genes were up-enriched at both 24 and 48 h in the A74 LPS vs. S comparison (Fig. 5C and G, Table S1). In addition, peroxidases related genes were significantly up-enriched in A6 and A74 LP vs. CK comparison combination at 24 h (Fig. 5D and Table S1), but only in A74 LP vs. CK comparison at 48 h (Fig. 5H and Table S1). Acid and other phosphatases related genes were significantly up-enriched in A6 LP vs. CK at 24 h and A74 LP vs. CK at 48 h (Fig. 5D and H, Table S1). Furthermore, we observed a significant up-enriched of the *MYB* domain transcription factor family exclusively at 24 h in the A74 LPS vs. S comparative combination (Fig. S2A). Additionally, the *GeBP* exhibited a significant up-enriched in the A74 LPS vs. S comparative combination at both 24 and 48 h (Fig. S2A and S2C). Phosphate transport related genes in the A6 LP vs. CK comparative combination displayed a significant down-enriched (Fig. S2B), which potentially contribute to abnormal phosphate uptake and transport in the LP environment. These gene categories played a role in A74’s ability to maintain high P-efficiency under salt stress.

### Co-expression network analysis and hub gene exploring by WGCNA

**Fig. 6:**
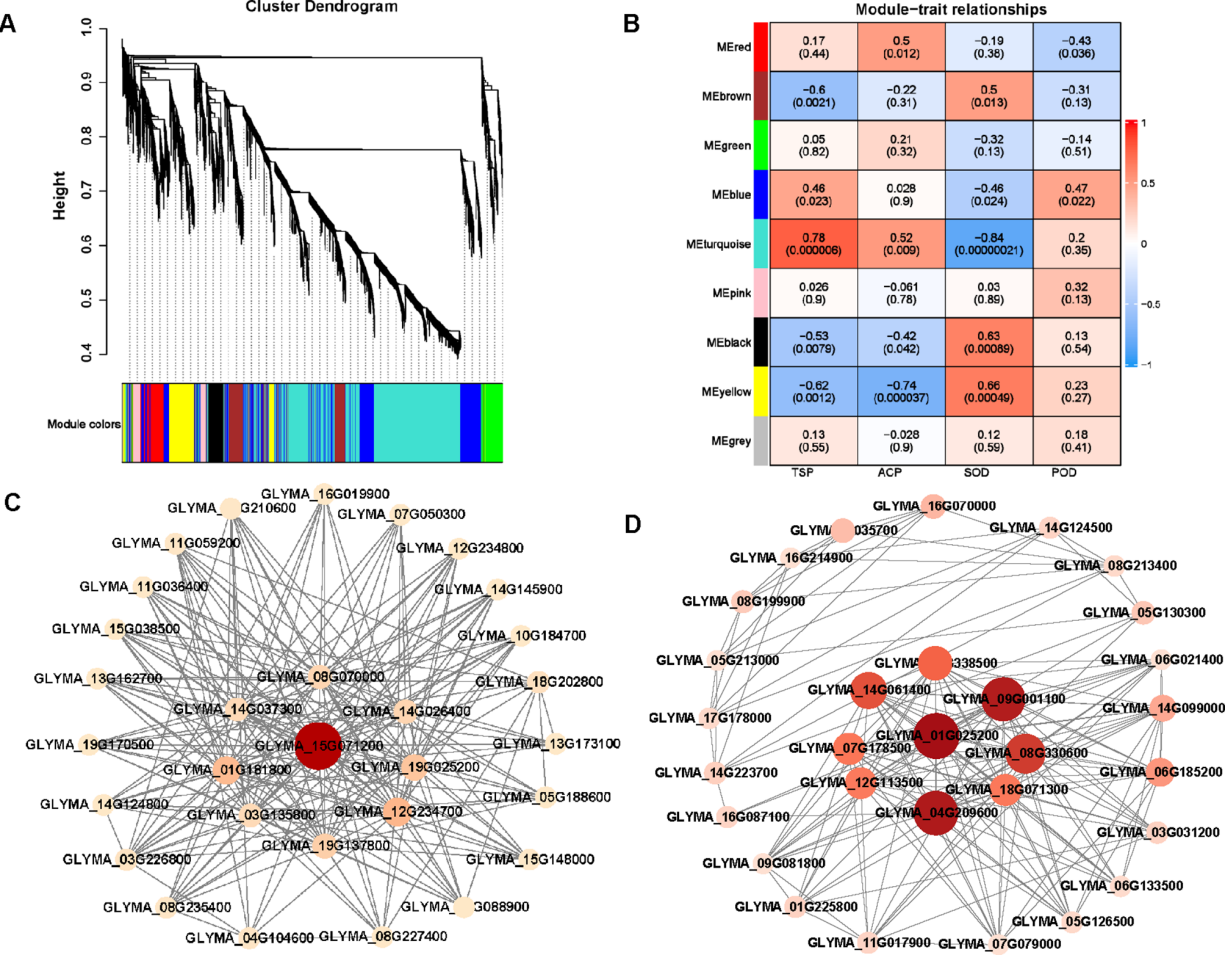
Weighted gene co-expression network analysis (WGCNA) of low phosphorus physiological indicators associated genes. (**A**) Gene dendrogram module colors showing 9 modules of co-expressed genes. A leaf represents each of the DEGs and a major tree branch represents each of the nine modules. (**B**) Module-traits relationships of different modules associated different traits of TSP, ACP, SOD and POD. Each row corresponds to a module characteristic gene (eigengene), and each column corresponds to a trait. The left panel shows nine modules. Each cell contains the corresponding correlation and *p* value. Visualization of key co-expression network of turquoise module with TSP and ACP (**C**) and yellow module with SOD (**D**) by Cytoscape. The size of node circle was positively correlated with the number of interacting genes

To study the correlation between traits and DEGs under different treatments, WGCNA analysis was conducted. Nine co-expression modules significantly correlated with physiological parameters were analyzed and identified (Fig. 6A). The module-traits analysis showed that TSP and ACP were positively correlated with the gene expression level in turquoise module, with correlation coefficients 0.78 and 0.52, and SOD was positively correlated with the gene expression level in yellow module with correlation coefficients 0.63 (Fig. 6B). These findings suggested that the turquoise module gene may be associated with increased ACP and TSP activity in A6 and A74, and the yellow module gene with increased SOD activity (Fig. 6B). At the *p*-value < 0.05 level, five modules were associated with TSP, four modules with ACP, five modules with SOD and two modules with POD (Fig. 6B).

Further, the turquoise module identified a total of 2,570 DEGS, while the yellow module identified 507 DEGs (Table S3). The turquoise co-expression network contained two hub genes in peroxisome: one citrate synthase gene (GLYMA_14G026400, *CS*) and one acyl-coenzyme A oxidase4 gene (GLYMA_18G202800, *ACX*) (Fig. 6C). Additionally, the hub genes in yellow co-expression network contained one cytokinin dehydrogenase 7 gene (GLYMA_14G099000, *CKXs*) and one two-component response regulator ARR2 gene (GLYMA_07G079000, *ARR2*) (Fig. 6D).

### GO and KEGG analysis of genes in turquoise and yellow module

**Fig. 7:**
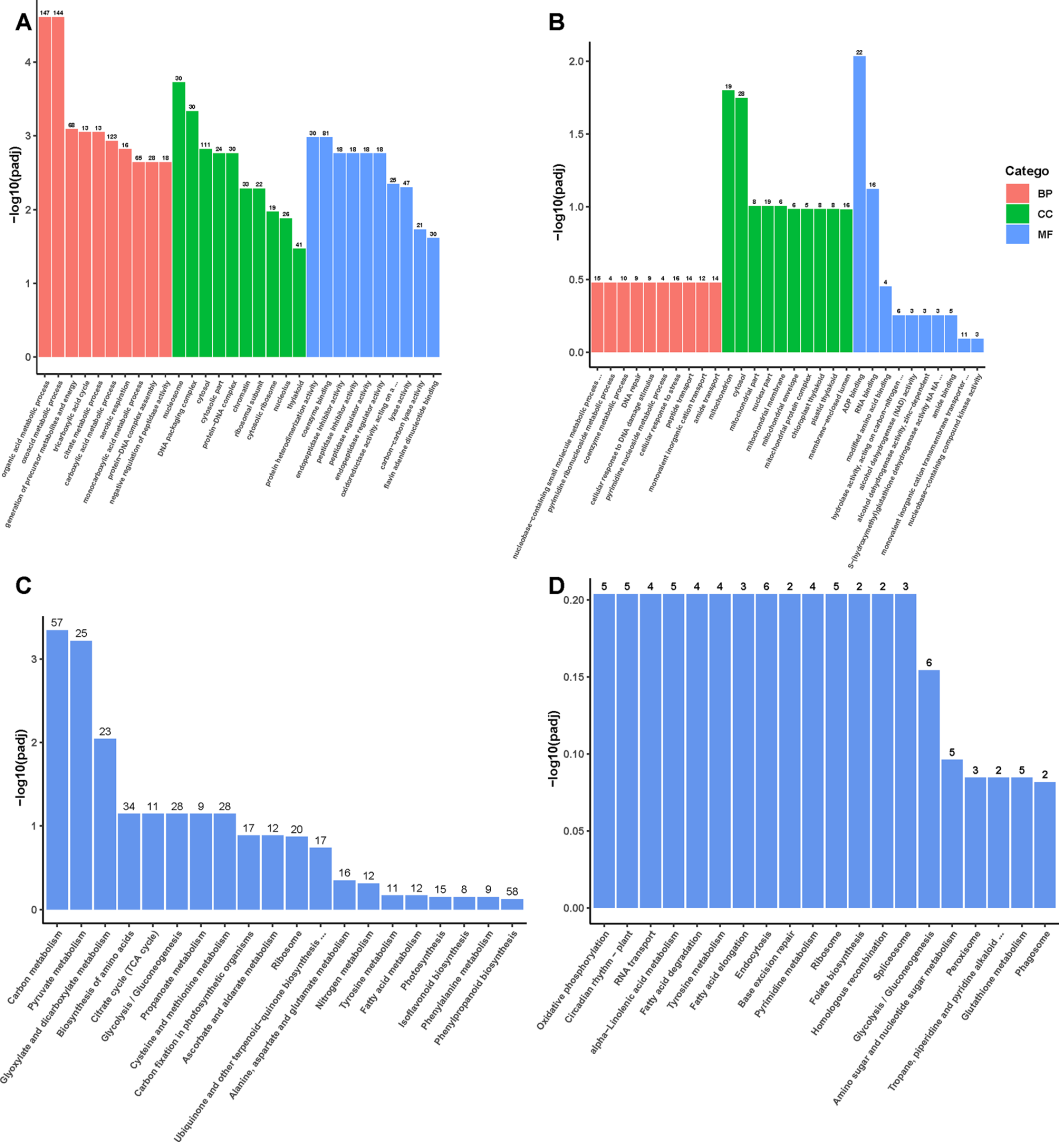
Gene Ontology enrichment analysis and KEGG pathway analysis of the DEGs. The enriched GO terms of MF, CC and BP from GO analysis of the turquoise(**A**) and yellow (**B**)modules DEGs. The high-enrichment KEGG pathways of the turquoise(**C**) and yellow (**D**) modules DEGs. *P* < 0.05

To be better explore the biological functions and pathways of the genes in turquoise and yellow modules, GO and KEGG analysis were performed. Each main functional category was further divided into ten smaller functional categories (Fig. 7A and B). Among the biological processes of turquoise module DEGs, organic acid metabolic process and oxoacid metabolic process were the two main functional categories that were enriched; nucleosome and DNA packaging complex were the functional categories of DEGs that were widely enriched in cellular components; the aggregation of DEGs in molecular function was relatively concentrated, mainly in protein heterodimerization activity and coenzyme binding (Fig. 7A). Regarding the biological processes of yellow module DEGs, most of them were enriched in cellular response to stress, nucleobase-containing small metabolic process; the major functional categories of DEGs enriched in cellular components were mitochondrion and cytosol; the aggregation of DEGs in molecular function was mainly manifested in ADP binding, and RNA binding (Fig. 7B). The KEGG analysis of DEGs in the turquoise module revealed that carbon metabolism was the most significantly enriched pathway (57 genes), followed by pyruvate metabolism (25 genes), and glyoxylate and dicarboxylate metabolism (23 genes) (Fig. 7C). In contrast, the yellow module DEGs were equally abundant in the first 14 pathways, with only the number of genes in each pathway varying. Endocytosis had the highest number of genes (6 genes) (Fig. 7D). These findings suggested that soybeans undergo changes in various metabolic pathways in response to LP and S related stress.

### RT-qPCR validation of selected deferentially expressed hub genes

**Fig. 8:**
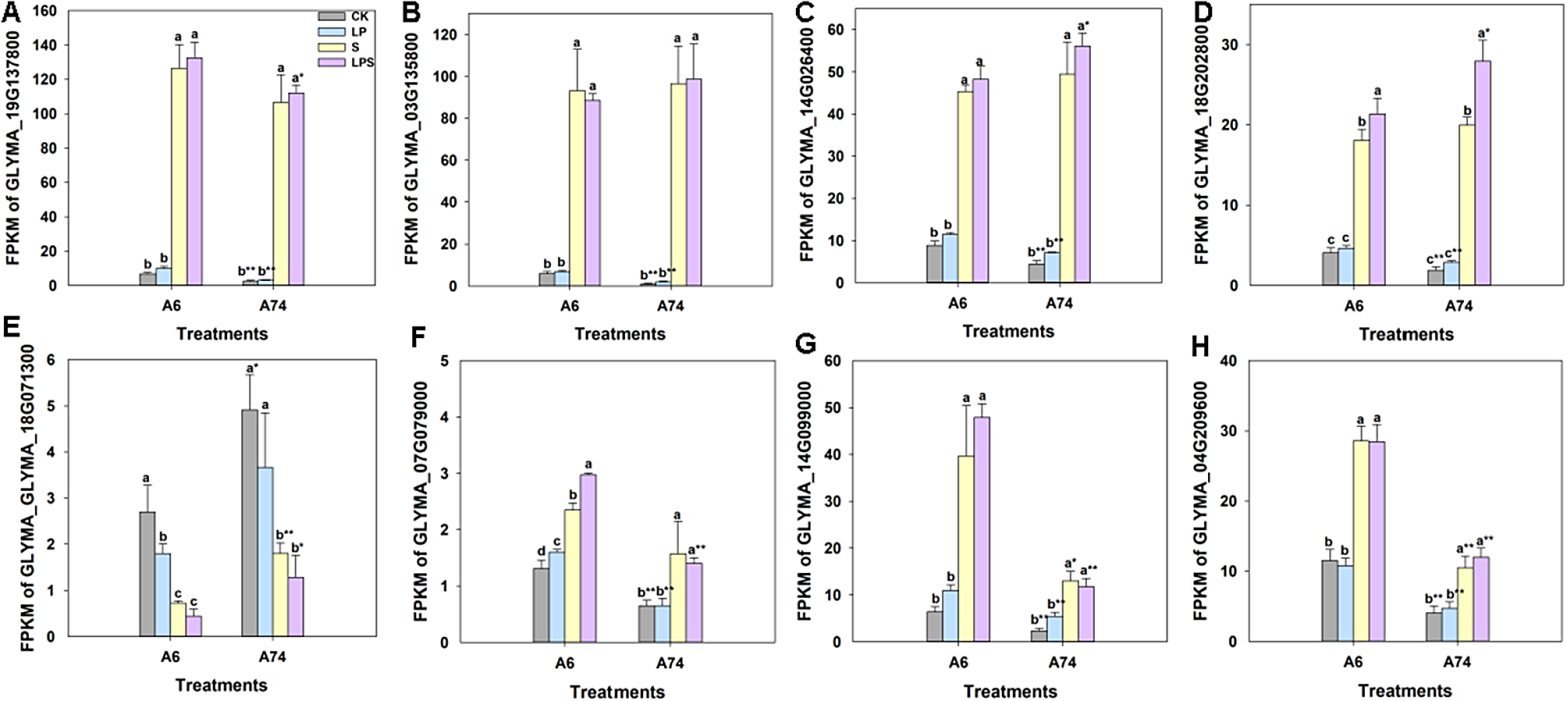
Transcript abundance of hub genes at 48 h in the turquoise and yellow modules. (**A**–**D**), hub genes from turquoise module. (**E**–**H**), hub genes from yellow module

To ensure the accuracy of hub gene identification based on the correlation between FPKM values and physiological parameters, it was necessary to confirm the FPKM values of these genes in the transcriptome data. In this study, the expression levels of eight hub genes from two WGCNA modules (turquoise and yellow) were determined using RT-qPCR analysis (Fig. S4 and Table. S4). These eight genes, consisting of seven up-regulated and one down-regulated genes in the A74 S vs. CK group (Fig. 8), exhibited different expression patterns. The RT-qPCR analysis confirmed that the expression patterns of these hub genes were consistent with their transcriptome FPKM values under the corresponding treatment (Fig. S4), indicating the reliability of the RNA-seq data.

## Discussion

Salinization is a leading cause of global agricultural soil degradation, regularly resulting in the loss of nutrient elements in saline-alkali soil [29]. To address this soil stress condition, it was important to explore the resistance mechanisms of salt-tolerant and P-efficient germplasms. Extensive research has been conducted on the transcriptional changes of soybeans in response to either P or salt stress alone [30–34]. Nevertheless, there remains a paucity of transcriptome studies examining the effects of LP and S combination stress on soybean. In this study, we investigated the distinct responses of two salt-tolerant germplasms: A74, known for its P-efficiency, and A6, recognized for its P-inefficiency, to LP conditions under salt stress. Our findings indicated that the growth and development of both germplasms were significantly impacted by low P and salt stress. However, under LP and LPS treatments, A74 demonstrated superior growth and development compared to A6, as depicted in Fig. 1A–E.

Energy is fundamental to all life activities, and ATP synthase plays a crucial role in the growth and development of plants, as highlighted by Liu et al. [35]. Upon PageMan analyzing, we observed that genes related to ATP synthesis were significantly up-regulated and enriched only in the A74 LP vs. CK comparison combination in the mitochondrial electron transport pathway (Fig. 5A and E). This up-regulation and enrichment of ATP synthesis-related genes served as a pivotal response strategy, endowing A74 with the essential energy required to mitigate the adverse impacts of P deficiency on its growth and development. In the comparative combination of A74 LP vs. CK at 48 h, it was found that the genes associated with alternative oxidase were significantly enriched and up-regulated (Fig. 5E). The pivotal role of alternative oxidase came into sharper focus, as this enzyme was empirically proven to enhance plants’ ability to absorb P in nutrient-deprived conditions, emphasizing the interplay between energy production and nutrient uptake [36–38]. These findings were also reflected in the phenotype, with A74 exhibiting significantly better growth and development under LP stress than A6 (Fig. 1A).

In response to P deficiency, plants evolved a series of intelligent adaptive mechanisms to improve P availability and increase its uptake efficiency, one of which was the production and secretion of acid phosphatase (ACP) [39, 40]. The increased expression and activity of *ACP* could improve the absorption and utilization efficiency of P [41]. Our study showed that A74 had higher P accumulation, P uptake efficiency, and ACP activity under both LP and LPS stress than A6 (Fig. 3A–C and Fig. S1A–D), suggesting it effectively acquired P resources in limited P conditions due to an enhanced P-efficiency mechanism. This mechanism allowed A74 to adapt better to P-scarce environment. P accumulation in A6 significantly decreased after 14 d of treatment under all stress conditions, while A74 only showed a decrease under LPS stress (Fig. 3A). This could be because A74, being P-efficient, took longer to adjust to LP and activate its P-efficient mechanism. The decline under LPS stemmed from increased injury under combined stress. This was reinforced by Fig. 1A, where plants under LP looked similar to CK, but those under LPS had notably poorer growth. Also, in the LP vs. CK comparison combination, genes related to acid and other phosphatases were found to be up-regulated in A6 at 24 h, and up-regulated in A74 at 48 h (Fig. 5D and H). Correspondingly, the ACP activity of A74 exhibited a significant increase under LP stress at 48 h (Fig. 2B). This finding revealed a more durable coping strategy of A74 to LP conditions. This strategy encompassed more effective P resource management to ensure a continuous supply of P and to mitigate the adverse effects of P deficiency on the plant, thus strengthening the adaptation of A74 under P-limited conditions. Moreover, phosphate transport-related genes were significantly down-enriched in the A6 LP vs. CK comparison combination at 24 h (Fig. S2B), which lead to abnormal P uptake and transport in LP environment, thus sensitizing A6 to exhibit sensitivity early in LP stress. The evidence indicated that *MYB* acted as a positive regulator of P transport [42]. Overexpression of *MYB* enhanced P uptake in plants [43], while its knockdown reduced phosphatase activity [44]. Our data showed that the *MYB* domain transcription factor family was notably up-enriched in the A74 LPS vs. S comparison combination at 24 h (Fig. S2A), which likely contributed to A74’s ability to sustain P-efficiency under combined stress and represented a key adaptive strategy of A74 to LPS stress.

Furthermore, our study had identified a module that was highly correlated with P absorption (Fig. 6C). The KEGG analysis of this module showed that DEGs were mainly enriched in carbon metabolism and pyruvate metabolism (Fig. 7C). Both metabolic processes played a pivotal role in facilitating plant energy supply responses [45, 46]. This reason was that they initiated a cascade of chemical reactions within the organism, consequently yielding additional energy to support the uptake of P by plants during the environmental stress. In this module, we identified two hub genes: *ACX* and *CS* (Fig. 6C). *ACX* played a crucial role in the β-oxidation of fatty acids, an energy-generating process in which the intermediate product acetyl-CoA was converted to citric acid by citrate synthase [47]. Citrate could enhance phosphate absorption under phosphate deficiency conditions [48], thus helping plants to survive in P-deficient environment. Similarly, the pyruvate metabolism also produces acetyl-CoA, subsequently leading to the production of citric acid which was a central part of carbon metabolism. These corresponded to the KEGG results of the module where the hub genes were located. In addition, *ACX* was involved in the synthesis of jasmonic acid [49], which could regulate phosphate homeostasis under phosphate deficiency [50, 51]. This finding further emphasized the important role of *ACX* in the uptake of P by plants. Under LPS stress, the expression levels of *CS* and *ACX* genes in A74 were significantly higher than those in A6 (Fig. 8C and D). This suggested A74 had a greater capacity for P uptake under salt stress and provided an explanation for the higher P-efficiency of A74 under salt stress. In summary, we posited that the genes identified through PageMan and WGCNA analyses contributed to A74’s high P-efficiency under salt stress.

Under LP stress, plants can be stimulated and induced to produce ROS, which activates their reactive oxygen scavenging system [52]. Major reactive oxygen scavenging enzymes, such as SOD and POD, can effectively clarify reactive oxygen species [53]. Furthermore, flavonoids were also capable of scavenging reactive oxygen species and controlling their accumulation [54]. Our study found that SOD activity increased significantly in A74 at 48 h in both LP vs. CK and LPS vs. S comparisons. In A74, POD activity also increased significantly in the LPS vs. S comparison at 24 and 48 h, while A6 showed no such changes (Fig. 2C and D). Moreover, the activity of genes encoding anthocyanin dioxygenase, chalone synthase, POD and glutathione S transferase significantly increased in the A74 LP vs. CK comparison combination (Fig. 5B, D, F and H). They played a pivotal role in regulating the scavenging of ROS, effectively shielding cells from ROS-induced damage [55–57]. As a result, this enhanced the adaptive resilience of A74 when subjected to LP-related condition. The genes related to receptor-like protein 7 (biotic. kinases) were found to be up-enriched only in the A74 LPS vs. S comparison combination (Fig. 5B and F). These genes play a crucial role in antioxidant stress [58], which indicating their activation under LPS stress to mitigate cellular damage.

Moreover, our research has identified a module that was highly associated with antioxidant potential (Fig. 6D). The GO analysis of this module demonstrated that its genes were predominantly enriched in biological processes related to cellular stress responses. This suggested that they likely assist cells in combating external environmental stress by regulating their antioxidant responses. *ARR2* and *CKXs* had been identified as hub genes in this module. *ARR2* acted as a transcription factor that was capable of reducing cytokinin signaling [59]. Meanwhile, cytokinin dehydrogenase played a crucial role as an enzyme responsible for degrading cytokinin [60].

Remarkably, the *GeBP* like transcription factor, known as a negative feedback regulator of *ARR* [61], exhibited significant up-enriched at both 24 hand 48 h in the A74 LP vs. CK comparison, which likely led to the suppression of A74 *ARR* expression in response to LP stress. This corresponded to our expression results that the expression levels of *ARR2* and *CKXs* genes were significantly lower in A74 than in A6 in response to LP and LPS stress (Fig. 8F and G). This differential gene expression resulted in increased cytokinin levels in A74 and decreased cytokinin levels in A6. In addition, cytokinin have been found to be associated with ROS clearance and a positive response to P deficiency [62–65]. These findings provided a possible explanation for why A74 was both salt tolerant and P-efficient, which was related to its unique gene expression and regulatory mechanisms.

## Conclusion

In this study, we revealed the differential response of two salt-tolerant soybean germplasms, A6 and A74, to low P stress through physiological and transcriptomic analysis, and investigated the reason why A74 maintained high P-efficiency under salt stress. We found that A74 achieved the goal of maintaining high P-efficiency under salt stress mainly through enhancements in its P uptake capacity and antioxidant capability. Of particular significance, the pinpointed hub genes potentially played a role in regulating A74’s P-efficiency during salt stress. Overall, this study provided detailed evidence to further understand the mechanism of salt-tolerant and P-efficiency of A74 at physiological and transcriptional levels, which provided new research perspectives and theoretical basis for P nutrient management and genetic improvement in salt-tolerant soybean.

## Materials and methods

### Plant materials and growth conditions

In this study, two soybean germplasms A6 (a salt-tolerance and P-inefficient germplasm) and A74 (a salt-tolerance and P-efficient germplasm) were employed. Both of these germplasms were sourced from the Soybean Research Institute of Shenyang Agricultural University.

Healthy soybean seeds of uniform size were selected for each germplasm. These seeds were disinfected with 1.0% sodium hypochlorite for 30 s, then rinsed three times with water. They were placed in 12 cm diameter plastic Petri dishes, each lined with sterilized filter paper. 20 mL distilled water was added in each petri dish, and pregerminated the seeds in an all-dark plant incubator at 28 °C. After 2 d, uniformly sprouted soybeans were transferred to a plastic box (310 mm × 290 mm × 180 mm) which containing 1/2 Hoagland nutrient solution. The 1/2 Hoagland nutrient solution components were given as follow: NH_4_H_2_PO_4_ (0.5 mM), KNO_3_ (2.5 mM), Ca (NO_3_)_2_.4H_2_O (2.5 mM), MgSO_4_.7H_2_O (1 mM), H_3_BO_3_ (23 µM), ZnSO_4_ .7H_2_O (0.38 µM), CuSO_4_·5H_2_O (0.16 µM), MnCl_2_ .4H_2_O (4.5 µM), H_2_MoO_4_ (0.2 µM), Fe-EDTA (25 µM). The plants were maintained in a plant incubator with an average day/night temperature of 28/24°C, 50% relative humidity, and a 15 h photoperiod. The nutrient solution was replaced every 3 d. Soybean seedlings at V2 growth stage, which is characterized by the development of two fully expanded trifoliate leaves, were chosen for transfer to hydroponic culture boxes.

### Treatments and experimental design

In order to identify the difference in P-efficiency between the two soybean germplasms, twenty-four uniformed V2 stage soybean seedlings were transplanted into four hydroponic culture boxes on March 9th, 2022. Control (CK), low phosphorus (LP), salt (S), low phosphorus + salt (LPS) were the four experimental treatments. The CK treatment solution retained the composition of 1/2 Hoagland nutrient solution; LP treatment solution decreased the P concentration to one-tenth of the original concentration (50 µM H_3_PO_4_); S treatment solution commenced with an initial addition of 50 mM NaCl to the 1/2 Hoagland solution. Subsequently, the NaCl concentration was incrementally increased by 50 mM every 3 d, ultimately reaching a final concentration of 200 mM. Similarly, the LPS treatment involved blending the LP nutrient solution with a gradual NaCl increment, starting at 50 mM and ascending by 50 mM increments every 3 d until reaching a final concentration of 200 mM. Each treatment contained three replications. Roots and leaves were harvested from the respective treatments upon the manifestation of significant phenotypic differences (March 23rd, 2022). Subsequently, these plant samples were utilized for further physiological investigations.

For transcriptome analysis, four experimental treatments were set up: control (CK), low phosphorus (LP), salt (S), low phosphorus + salt (LPS). Forty-eight uniformed seedlings of A6 and A74 were arranged into four hydroponic boxes on June 6th, 2022. The boxes contained different solutions: CK (1/2 Hoagland nutrient solution), LP (P concentration decreased to one-tenth of the original concentration, equivalent to 50 µM H_3_PO_4_), S (1/2 Hoagland nutrient solution supplemented with 200 mM NaCl), and LPS (LP solution further supplemented with 200 mM NaCl). The plants were then treated for 24 and 48 h respectively. Each treatment contained three replications. Roots and leaves were collected for physiological and molecular analysis.

## Methods

### Growth and biomass assessment

The assessment of the relative growth rate, root length, and biomass of soybean seedlings was conducted during a trial period starting on March 9th, 2022. Plant height was measured at the beginning and end of the trial (March 23rd), with the change in height (∆H) used to estimate the relative growth rate of the shoot using the formula ∆H/14. Root length was also measured on March 23rd. For biomass analysis, both shoots and roots were harvested on the same day, dried on absorbent paper, and their fresh weight was precisely measured on a 1/10,000 scale. They were then heated initially at 105 °C for 30 min and dried at 80 °C until a constant weight was obtained for dry weight determination.

### Biochemical analysis

For biochemical parameters, approximately 0.2 g of dry weight from shoots and roots, from three biological replicates, were digested in H_2_SO_4_, boiled, and further processed at 370 °C until the solution cleared for P content analysis using an automatic discrete analyzer (Smartchem 200; AMS Alliance, Guidonia, Rome, Italy). Acid phosphatase levels were determined using kits from Suzhou Keming Biotechnology Co., LTD. Total soluble protein content was measured by a modified dye-binding assay [66], with absorbance read at 595 nm using a spectrophotometer (UV-2600, UNICO Instruments Co., Ltd., Shanghai, China), and bovine serum albumin as the standard. Furthermore, the activities of superoxide dismutase (SOD) and peroxidase (POD) enzymes were measured according to previously described methods [20, 67].

### Transcriptome analysis

Root samples of 24 and 48 h were subjected to transcriptome analysis. Each treatment comprised three replicates, resulting in a total of 48 root samples for RNA sequencing. Trizol reagent (Invitrogen, America) was used to isolate total RNA from about 0.1 g samples of roots. DNase I was used to eliminate contaminating genomic DNA from RNA. A UV spectrophotometry NanoDrop was used to examine the RNA concentration and purity (NanoDropND-1000 UV-Vis spectrophotometer). The overall quantity and integrity of the RNA were further evaluated using the RNA Nano 6000 Assay Kit of the Bioanalyzer 2100 system (Agilent Technologies, CA, USA). To specifically select cDNA fragments with a length of 370–420 bp, the library fragments were purified with AMPure XP system (Bechman Coulter, Beverly, USA). Then PCR amplification, the PCR product was further purified by AMPure XP beads, and the library was finally obtained. Agilent 2100 bioanalyzer was used to test the quality of library. After the library was qualified, the different libraries were pooling according to the effective concentration and the target amount of data off the machine, then being sequenced by the Illumina NovaSeq 6000. The end reading of 150 bp pairing was generated. Clean reads were obtained by removing those containing adapters, N bases, and low-quality reads from the raw data. The expression of transcripts was analyzed by the cufflinks program and their expression distribution was estimated based on FPKM value. We compare the expression changes in the CK, LP, S, LPS treated samples to identify DEGs. The significance of differential gene expression was evaluated on the basis of the following thresholds: log2foldchange value ≥ 1 or ≤ − 1 and FDR (false discovery rate) ≤ 0.05 and FPKM value ≥ 1.

### PageMan analysis

The log2foldchange of A6 LP vs. CK, A74 LP vs. CK, A6 LPS vs. S, A74 LPS vs. S at 24 and 48 h were imported into PageMan and the over-representation in all of the treatments were compared [68]. The PageMan statistical analysis was used to predict significant effect of BINs. Wilcoxon test was applied to analyze data. Significant differences of BINs were defined in terms of a *p*-value < 0.05. Blue color indicated a significant reduction of up-regulated genes and red color indicated a significant enrichment of up-regulated genes.

### Weighted correlation network analysis

Weighted correlation network analysis (WGCNA) is a systematic biological method utilized to describe the gene association patterns among different samples. In this study, genes were analyzed using WGCNA against the physiological parameters from the same samples, employing hypergeometric tests [69]. The FPKM values were initially normalized by square root transformation, and the cutoff for significant enrichment was set at FDR < 0.05. The automatic one-step method with default settings was applied to conduct network construction and module detection. The association of modules with each physiological parameter of 48 samples were determined by the calculated module eigengene value. Modules demonstrating notable relevance to physiological parameters were depicted using Cytoscape for visualization [70].

### GO and KEGG analysis

Gene Ontology (GO) enrichment analysis and Kyoto Encyclopedia of Genes and Genomes (KEGG) pathway were performed using TBtools [71]. *P* < 0.05 was set as the threshold for both analyses.

### Analysis of gene expression by RT-qPCR

Trizol reagent (Invitrogen, America) was used to isolated total RNA from about 0.1 g samples of roots from three biological replicates at all two time points. The RNA was then treated with DNase I to remove contaminating genomic DNA. The RNA concentration and purity were assessed using a UV spectrophotometry NanoDrop (NanoDropND-1000 UV-Vis spectrophotometer).

2.5 µg RNA was reverse transcribed to cDNA using a Hifair III 1st Strand cDNA Synthesis SuperMix for qPCR with genome-DNA-removing enzyme (Yesen, Nanjing, China). The qPCR was performed on QuantStudio 6 (ABI, Forster City, CA, USA) detection system using SYBR green PCR mix (Takara, RR420A, Shika, Japan). The real-time PCR program was as follows: 95 °C for 5 min; 40 cycled of 95 °C for 10 s and 60 °C for 30 s. The primers used were detailed in Table S4. Primer specificity was checked by BLASTN searches against sequences in the soybean genome database (Phytozome) with the designed primers as queries, melt curve analysis, and agarose gel electrophoresis.

### Statistical analysis

All treatments had three replicates. All the data was subjected to analysis of variance (AVOVA) with the Duncan’s multiple range tests means at a significant level of *P* < 0.05 using the statistical package SPSS 16.0, Origin Pro 9.0 and Excel 2019 for Windows.

### Electronic supplementary material

Below is the link to the electronic supplementary material.

**Supplementary Material 1: Figure S1** The phosphorus accumulation and uptake efficiency of shoot and root

**Supplementary Material 2: Figure S2** PageMan analysis of DEGs related to transcription factors and transporters under different treatments. Transcription factors (A, C), transporters (B, D)

**Supplementary Material 3: Figure S3** The complete view of enriched gene categories using PageMan analysis

**Supplementary Material 4: Figure S4** Relative expression levels of genes in the turquoise and yellow modules. A-D, genes from turquoise module. E-H, genes from yellow module

**Supplementary Material 5: Table S1** The list of time-specific enriched sub-bins of DEGs using MapMan systems. The genes from different enriched categories at each time point are given in log2 scale, and can be distinguished by the column headers

**Supplementary Material 6: Table S2** The physiological index used for WGCNA analysis and hub genes involved in turquoise and yellow modules

**Supplementary Material 7: Table S3** The number of genes in different modules

**Supplementary Material 8: Table S4** The primes of genes in turquoise and yellow modules used for RT-qPCR

**Supplementary Material 9: Table S5** All the identified DEGs

Supplementary Material 10

## Data Availability

The raw RNAseq data has been successfully uploaded to NCBI and the accession number for our submission is: PRJNA1030137. The materials used during the current study are available from the corresponding author on reasonable request.

## Abbreviations

CK: control
LP: low phosphorus
S: salt
P: phosphorus
LPS: low phosphorus + salt
ACP: acid phosphatase
TSP: total soluble protein
SOD: superoxide dismutase
POD: peroxidase
ROS: reactive oxygen species
CS: Citrate synthase gene
ACX: acyl-coenzyme A oxidase
CKXs: cytokinin dehydrogenase
WGCNA: Weighted correlation network analysis

## References

[1] Lv S, Wang D, Jiang P, Jia W, Li Y (2021). Variation of PHT families adapts salt cress to phosphate limitation under salinity. Plant Cell Environ.

[2] Giri J, Bhosale R, Huang G, Pandey BK, Parker H, Zappala S (2018). Rice auxin influx carrier OsAUX1 facilitates root hair elongation in response to low external phosphate. Nat Commun.

[3] Peng W, Wu W, Peng J, Li J, Lin Y, Wang Y (2018). Characterization of the soybean GmALMT family genes and the function of GmALMT5 in response to phosphate Starvation. J Integr Plant Biol.

[4] Mo X, Zhang M, Liang C, Cai L, Tian J (2019). Integration of metabolome and transcriptome analyses highlights soybean roots responding to phosphorus deficiency by modulating phosphorylated metabolite processes. Plant Physiol Biochem.

[5] Yamaji N, Takemoto Y, Miyaji T, Mitani-Ueno N, Yoshida KT, Ma JF (2017). Reducing phosphorus accumulation in rice grains with an impaired transporter in the node. Nature.

[6] Chu S, Zhang X, Yu K, Lv L, Sun C, Liu X (2020). Genome-wide analysis reveals dynamic epigenomic differences in soybean response to low-phosphorus stress. Int J Mol Sci.

[7] Yuan H, Liu D (2008). Signaling components involved in plant responses to phosphate Starvation. J Integr Plant Biol.

[8] Fixen PE, Johnston AM (2012). World fertilizer nutrient reserves: a view to the future. J Sci Food Agric.

[9] Han Y, White PJ, Cheng L (2022). Mechanisms for improving phosphorus utilization efficiency in plants. Ann Bot.

[10] Cong WF, Suriyagoda LDB, Lambers H (2020). Tightening the Phosphorus cycle through phosphorus-efficient crop genotypes. Trends Plant Sci.

[11] Cai Z, Cheng Y, Xian P, Ma Q, Wen K, Xia Q. er al. Acid phosphatase gene GmHAD1 linked to low phosphorus tolerance in soybean, through fine mapping. Theor Appl Genet. 2018;131(8):1715–1728. 10.1007/s00122-018-3109-3 29754326

[12] Zhang D, Song H, Cheng H, Hao D, Wang H, Kan G (2014). The acid phosphatase-encoding gene GmACP1 contributes to soybean tolerance to low-phosphorus stress. PLoS Genet.

[13] Haling RE, Brown LK, Bengough AG, Young IM, Hallett PD, White PJ (2013). Root hairs improve root penetration, root-soil contact, and phosphorus acquisition in soils of different strength. J Exp Bot.

[14] Lambers H, Shane MW, Cramer MD, Pearse SJ, Veneklaas EJ (2006). Root structure and functioning for efficient acquisition of phosphorus: matching morphological and physiological traits. Ann Bot.

[15] White PJ, George TS, Gregory PJ, Bengough AG, Hallett PD, McKenzie BM (2013). Matching roots to their environment. Ann Bot.

[16] Vance CP, Uhde-Stone C, Allan DL (2003). Phosphorus acquisition and use: critical adaptations by plants for securing a nonrenewable resource. New Phytol.

[17] de Bang TC, Husted S, Laursen KH, Persson DP, Schjoerring JK (2021). The molecular-physiological functions of mineral macronutrients and their consequences for deficiency symptoms in plants. New Phytol.

[18] van Zelm E, Zhang Y, Testerink C (2020). Salt tolerance mechanisms of plants. Annu Rev Plant Biol.

[19] Zhang Z, Niu B, Li X, Kang X, Wan H, Shi X (2022). Inversion of soil salinity in China’s Yellow River Delta using unmanned aerial vehicle multispectral technique. Environ Monit Assess.

[20] Yan F, Wei H, Ding Y, Li W, Liu Z, Chen L (2021). Melatonin regulates antioxidant strategy in response to continuous salt stress in rice seedlings. Plant Physiol Biochem.

[21] Loudari A, Mayane A, Zeroual Y, Colinet G, Oukarroum A (2022). Photosynthetic performance and nutrient uptake under salt stress: Differential responses of wheat plants to contrasting phosphorus forms and rates. Front Plant Sci.

[22] Zahedi SM, Hosseini MS, Abadía J, Marjani M (2020). Melatonin foliar sprays elicit salinity stress tolerance and enhance fruit yield and quality in strawberry (Fragaria × ananassa Duch). Plant Physiol Biochem.

[23] Lu Q, Ge G, Sa D, Wang Z, Hou M, Jia YS (2021). Effects of salt stress levels on nutritional quality and microorganisms of alfalfa-influenced soil. PeerJ.

[24] Sadji-Ait Kaci H, Chaker- Haddadj A, Aid F (2018). Enhancing of symbiotic efficiency and salinity tolerance of chickpea by phosphorus supply. Acta Agriculturae Scandinavica Section B—Soil & Plant Science.

[25] Tang H, Niu L, Wei J, Chen X, Chen Y (2019). Phosphorus Limitation Improved Salt Tolerance in Maize through tissue Mass Density increase, Osmolytes Accumulation, and na + uptake inhibition. Front Plant Sci.

[26] Nieman RH, Clark RA (1976). Interactive effects of salinity and phosphorus nutrition of the concentrations of phosphate and phosphate esters in mature photosynthesizing corn leaves. Plant Physiol.

[27] Kumar V, Singh TR, Hada A, Jolly M, Ganapathi A, Sachdev A (2015). Probing phosphorus efficient low phytic acid content soybean genotypes with Phosphorus Starvation in Hydroponics Growth System. Appl Biochem Biotechnol.

[28] Li H, Xu L, Li J, Lyu X, Li S, Wang C (2022). Multi-omics analysis of the regulatory effects of low-phosphorus stress on phosphorus transport in soybean roots. Front Plant Sci.

[29] Bargaz A, Lyamlouli K, Chtouki M, Zeroual Y, Dhiba D (2018). Soil Microbial resources for improving fertilizers efficiency in an Integrated Plant Nutrient Management System. Front Microbiol.

[30] Gao S, Guo R, Liu Z, Hu Y, Guo J, Sun M (2023). Integration of the transcriptome and metabolome reveals the mechanism of resistance to low phosphorus in wild soybean seedling leaves. Plant Physiol Biochem.

[31] Hu J, Zhuang Y, Li X, Li X, Sun C, Ding Z (2022). Time-series transcriptome comparison reveals the gene regulation network under salt stress in soybean (Glycine max) roots. BMC Plant Biol.

[32] Liu X, Yang X, Zhang B (2021). Transcriptome analysis and functional identification of GmMYB46 in soybean seedlings under salt stress. PeerJ.

[33] Mo X, Liu G, Zhang Z, Lu X, Liang C, Tian J (2022). Mechanisms underlying soybean response to Phosphorus Deficiency through Integration of Omics Analysis. Int J Mol Sci.

[34] Yang T, Yang S, Chen Z, Tan Y, Bol R, Duan H (2022). Global transcriptomic analysis reveals candidate genes associated with different phosphorus acquisition strategies among soybean germplasms. Front Plant Sci.

[35] Liu T, Arsenault J, Vierling E, Kim M (2021). Mitochondrial ATP synthase subunit d, a component of the peripheral stalk, is essential for growth and heat stress tolerance in Arabidopsis thaliana. Plant J.

[36] Del-Saz NF, Romero-Munar A, Cawthray GR, Palma F, Aroca R, Baraza E (2018). Phosphorus concentration coordinates a respiratory bypass, synthesis and exudation of citrate, and the expression of high-affinity phosphorus transporters in Solanum lycopersicum. Plant Cell Environ.

[37] Florez-Sarasa I, Lambers H, Wang X, Finnegan PM, Ribas-Carbo M (2014). The alternative respiratory pathway mediates carboxylate synthesis in white lupin cluster roots under phosphorus deprivation. Plant Cell Environ.

[38] Sieger SM, Kristensen BK, Robson CA, Amirsadeghi S, Eng EW, Abdel-Mesih A (2005). The role of alternative oxidase in modulating carbon use efficiency and growth during macronutrient stress in Tobacco cells. J Exp Bot.

[39] del Pozo JC, Allona I, Rubio V, Leyva A, de la Peña A, Aragoncillo C (1999). A type 5 acid phosphatase gene from Arabidopsis thaliana is induced by phosphate Starvation and by some other types of phosphate mobilising/oxidative stress conditions. Plant J.

[40] Hur YJ, Lee HG, Jeon EJ, Lee YY, Nam MH, Yi G (2007). A phosphate starvation-induced acid phosphatase from Oryza sativa: phosphate regulation and transgenic expression. Biotechnol Lett.

[41] Deng S, Li J, Du Z, Wu Z, Yang J, Cai H (2022). Rice ACID PHOSPHATASE 1 regulates pi stress adaptation by maintaining intracellular pi homeostasis. Plant Cell Environ.

[42] Valdés-López O, Arenas-Huertero C, Ramírez M, Girard L, Sánchez F, Vance CP (2008). Essential role of MYB transcription factor: PvPHR1 and microRNA: PvmiR399 in phosphorus-deficiency signalling in common bean roots. Plant Cell Environ.

[43] Nilsson L, Müller R, Nielsen TH (2007). Increased expression of the MYB-related transcription factor, PHR1, leads to enhanced phosphate uptake in Arabidopsis thaliana. Plant Cell Environ.

[44] Kumar Sharma A, Mühlroth A, Jouhet J, Maréchal E, Alipanah L, Kissen R (2020). The myb-like transcription factor phosphorus Starvation response (PtPSR) controls conditional P acquisition and remodelling in marine microalgae. New Phytol.

[45] Chen WW, Freinkman E, Wang T, Birsoy K, Sabatini DM (2016). Absolute quantification of Matrix metabolites reveals the dynamics of mitochondrial metabolism. Cell.

[46] Pascal R, Boiteau L (2011). Energy flows, metabolism and translation. Philos Trans R Soc Lond B Biol Sci.

[47] Pracharoenwattana I, Cornah JE, Smith SM (2005). Arabidopsis peroxisomal citrate synthase is required for fatty acid respiration and seed germination. Plant Cell.

[48] López-Bucio J, de La Vega OM, Guevara-García A, Herrera-Estrella L (2000). Enhanced phosphorus uptake in transgenic Tobacco plants that overproduce citrate. Nat Biotechnol.

[49] Xin Z, Chen S, Ge L, Li X, Sun X (2019). The involvement of a herbivore-induced acyl-CoA oxidase gene, CsACX1, in the synthesis of jasmonic acid and its expression in flower opening in tea plant (Camellia sinensis). Plant Physiol Biochem.

[50] Deepika SA (2021). Expression dynamics indicate the role of jasmonic acid biosynthesis pathway in regulating macronutrient (N, P and K^+^) deficiency tolerance in rice (Oryza sativa L). Plant Cell Rep.

[51] Tao Y, Huang J, Jing HK, Shen RF, Zhu XF (2022). Jasmonic acid is involved in root cell wall phosphorus remobilization through the nitric oxide dependent pathway in rice. J Exp Bot.

[52] Li C, Li K, Zheng M, Liu X, Ding X, Gai J (2021). Gm6PGDH1, a cytosolic 6-Phosphogluconate dehydrogenase, enhanced tolerance to phosphate Starvation by improving Root System Development and modifying the antioxidant system in soybean. Front Plant Sci.

[53] Shin R, Berg RH, Schachtman DP (2005). Reactive oxygen species and root hairs in Arabidopsis root response to nitrogen, phosphorus and potassium deficiency. Plant Cell Physiol.

[54] Dias MC, Pinto DCGA, Silva AMS (2021). Plant flavonoids: chemical characteristics and biological activity. Molecules.

[55] Dvořák P, Krasylenko Y, Zeiner A, Šamaj J, Takáč T (2021). Signaling toward reactive oxygen species-scavenging enzymes in plants. Front Plant Sci.

[56] Lijuan C, Huiming G, Yi L, Hongmei C (2015). Chalcone synthase EaCHS1 from Eupatorium adenophorum functions in salt stress tolerance in Tobacco. Plant Cell Rep.

[57] Zhou Y, Mumtaz MA, Zhang Y, Yang Z, Hao Y, Shu H (2022). Response of anthocyanin biosynthesis to light by strand-specific transcriptome and miRNA analysis in Capsicum annuum. BMC Plant Biol.

[58] Pitorre D, Llauro C, Jobet E, Guilleminot J, Brizard JP, Delseny M (2010). RLK7, a leucine-rich repeat receptor-like kinase, is required for proper germination speed and tolerance to oxidative stress in Arabidopsis thaliana. Planta.

[59] Kim K, Hwang I (2012). Attenuation of cytokinin signaling via proteolysis of a type-B response regulator. Plant Signal Behav.

[60] Du Y, Zhang Z, Gu Y, Li W, Wang W, Yuan X (2023). Genome-wide identification of the soybean cytokinin oxidase/dehydrogenase gene family and its diverse roles in response to multiple abiotic stress. Front Plant Sci.

[61] Chevalier F, Perazza D, Laporte F, Le Hénanff G, Hornitschek P, Bonneville JM (2008). GeBP and GeBP-like proteins are noncanonical leucine-zipper transcription factors that regulate cytokinin response in Arabidopsis. Plant Physiol.

[62] Jiang L, Liu C, Cao H, Chen Z, Yang J, Cao S (2019). The role of cytokinin in selenium stress response in Arabidopsis. Plant Sci.

[63] Niu YF, Chai RS, Jin GL, Wang H, Tang CX, Zhang YS (2013). Responses of root architecture development to low phosphorus availability: a review. Ann Bot.

[64] Xu Y, Burgess P, Zhang X, Huang B (2016). Enhancing cytokinin synthesis by overexpressing ipt alleviated drought inhibition of root growth through activating ROS-scavenging systems in Agrostis stolonifera. J Exp Bot.

[65] Yan H, Wang Y, Chen B, Wang W, Sun H, Sun H (2022). OsCKX2 regulates phosphate deficiency tolerance by modulating cytokinin in rice. Plant Sci.

[66] Goldring JPD (2019). Measuring protein concentration with Absorbance, Lowry, Bradford Coomassie Blue, or the Smith Bicinchoninic Acid Assay before Electrophoresis. Methods Mol Biol.

[67] Hu T, Li HY, Zhang XZ, Luo HJ, Fu JM (2011). Toxic effect of NaCl on ion metabolism, antioxidative enzymes and gene expression of perennial ryegrass. Ecotoxicol Environ Saf.

[68] Usadel B, Nagel A, Steinhauser D, Gibon Y, Bläsing OE, Redestig H (2006). PageMan: an interactive ontology tool to generate, display, and annotate overview graphs for profiling experiments. BMC Bioinformatics.

[69] Langfelder P, Horvath S (2008). WGCNA: an R package for weighted correlation network analysis. BMC Bioinformatics.

[70] Maere S, Heymans K, Kuiper M (2005). BiNGO: a Cytoscape plugin to assess overrepresentation of gene ontology categories in biological networks. Bioinformatics.

[71] Chen C, Chen H, Zhang Y, Thomas HR, Frank MH, He Y (2020). TBtools: an integrative Toolkit developed for interactive analyses of big Biological Data. Mol Plant.

