# Supplementary figures and images for: Comparative physiological and transcriptomic analysis of two salt-tolerant soybean germplasms response to low phosphorus stress: role of phosphorus uptake and antioxidant capacity
Source: BMC Plant Biol. 2023 Dec 20;23:662. doi: 10.1186/s12870-023-04677-y (PMC10731862; doi:10.1186/s12870-023-04677-y)

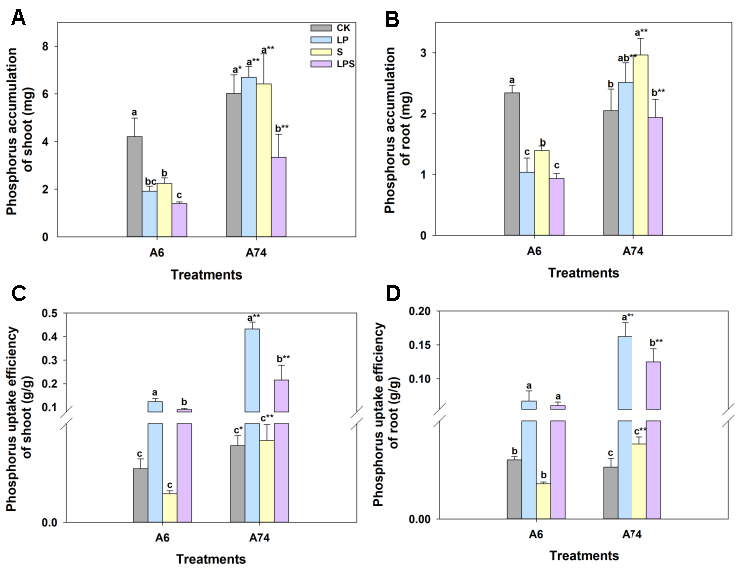

Supplement: Supplementary file 1 — Supplementary Material 1: Figure S1 The phosphorus accumulation and uptake efficiency of shoot and root [file 12870_2023_4677_MOESM1_ESM.png]

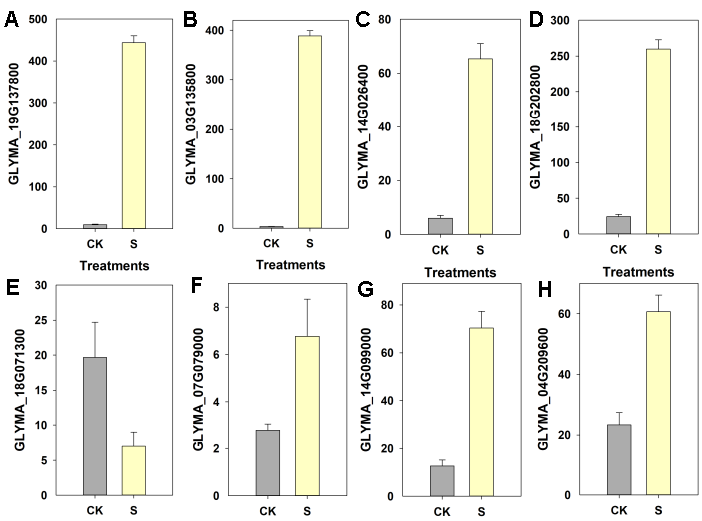

Supplement: Supplementary file 4 — Supplementary Material 4: Figure S4 Relative expression levels of genes in the turquoise and yellow modules. A-D, genes from turquoise module. E-H, genes from yellow module [file 12870_2023_4677_MOESM4_ESM.png]

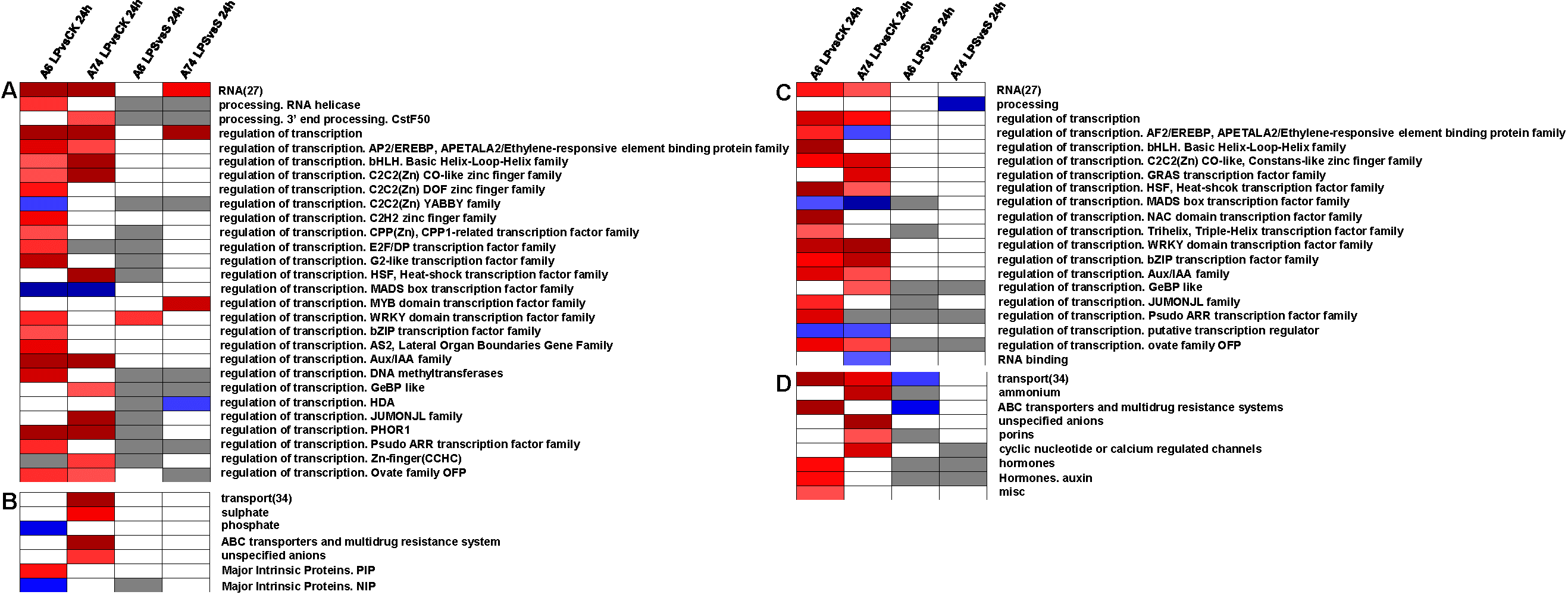

Supplement: Supplementary file 5 — Supplementary Material 5: Table S1 The list of time-specific enriched sub-bins of DEGs using MapMan systems. The genes from different enriched categories at each time point are given in log2 scale, and can be distinguished by the column headers [file 12870_2023_4677_MOESM5_ESM.png]

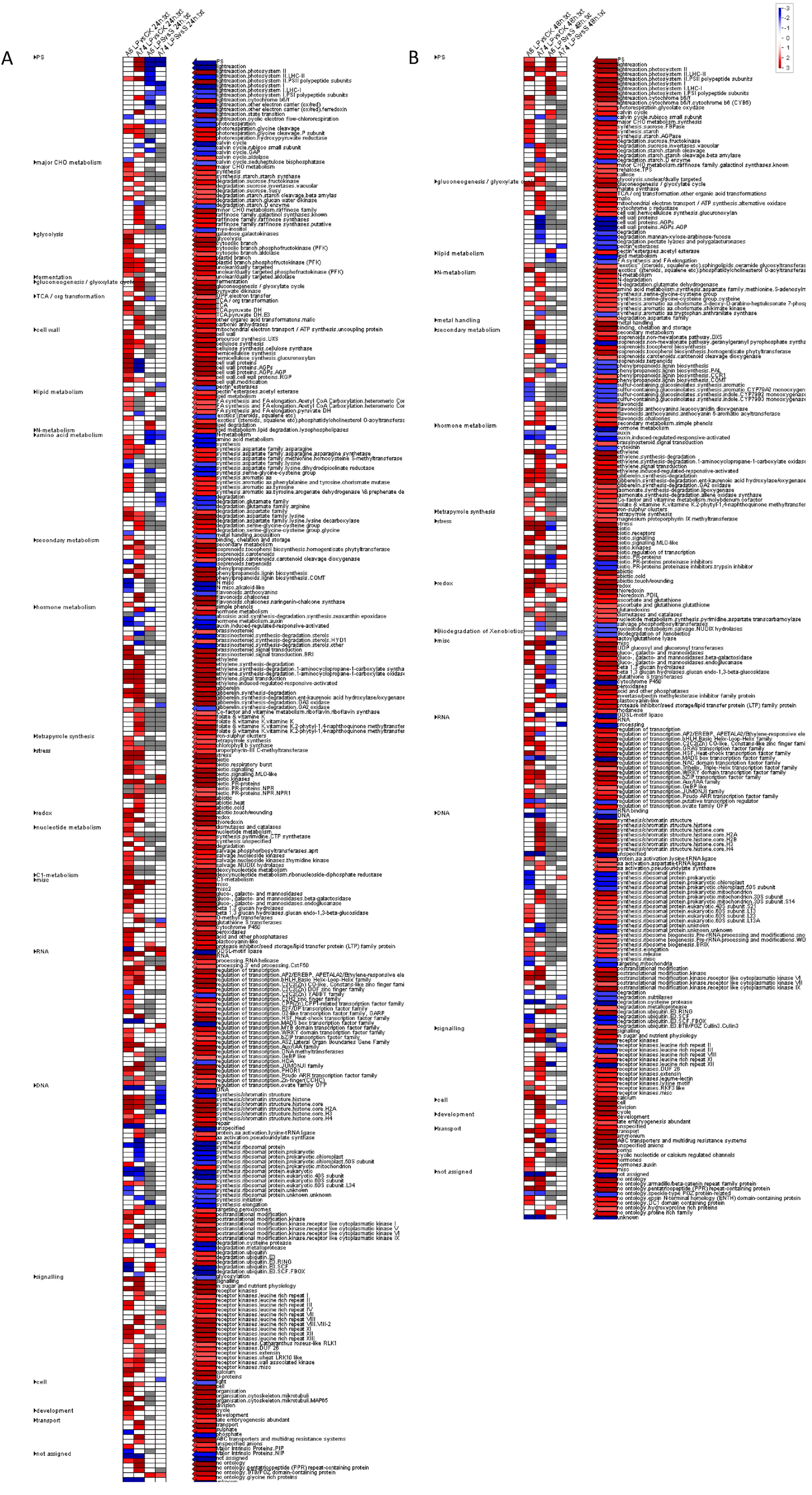

Supplement: Supplementary file 8 — Supplementary Material 8: Table S4 The primes of genes in turquoise and yellow modules used for RT-qPCR [file 12870_2023_4677_MOESM8_ESM.png]
