# Supplementary material for: Comparative physiological and transcriptomic analysis of two salt-tolerant soybean germplasms response to low phosphorus stress: role of phosphorus uptake and antioxidant capacity
Source: BMC Plant Biol. 2023 Dec 20;23:662. doi: 10.1186/s12870-023-04677-y (PMC10731862; doi:10.1186/s12870-023-04677-y)
Supplement: Supplementary file 10 — Supplementary Material 10 [file 12870_2023_4677_MOESM10_ESM.docx]

**Supplementary Information**

**Figure** **S1** The phosphorus accumulation and uptake efficiency of shoot and root.

**Figure** **S2** PageMan analysis of DEGs related to transcription factors and transporters under different treatments. Transcription factors (A, C), transporters (B, D).

**Figure** **S3** The complete view of enriched gene categories using PageMan analysis.

**Figure** **S4** Relative expression levels of genes in the turquoise and yellow modules. A-D, genes from turquoise module. E-H, genes from yellow module.

**Table S1** The list of time-specific enriched sub-bins of DEGs using MapMan systems. The genes from different enriched categories at each time point are given in log2 scale, and can be distinguished by the column headers.

**Table S2** The physiological index used for WGCNA analysis and hub genes involved in turquoise and yellow modules.

**Table S3** The number of genes in different modules.

**Table S4** The primes of genes in turquoise and yellow modules used for RT-qPCR.

**Table S5** All the identified DEGs.
